# A Novel Mutation of *GARS* in a Chinese Family With Distal Hereditary Motor Neuropathy Type V

**DOI:** 10.3389/fneur.2018.00571

**Published:** 2018-07-23

**Authors:** Xueying Yu, Bin Chen, Hefei Tang, Wei Li, Ying Fu, Zaiqiang Zhang, Yaping Yan

**Affiliations:** ^1^Department of Neurology, China National Clinical Research Center for Neurological Diseases, Beijing Tiantan Hospital, Capital Medical University, Beijing, China; ^2^Key Laboratory of the Ministry of Education for Medicinal Resources and Natural Pharmaceutical Chemistry, National Engineering Laboratory for Resource Development of Endangered Crude Drugs in Northwest of China, College of Life Sciences, Shaanxi Normal University, Xi'an, China

**Keywords:** dHMN-V, CMT2D, Novel mutation, GARS gene, silico analysis

## Abstract

Glycyl-tRNA synthetase (*GARS)* gene mutations have been reported to be associated with Charcot-Marie-Tooth disease 2D and distal hereditary motor neuropathy type V (dHMN-V). In this study, we report a novel *GARS* mutation in a Chinese family with dHMN-V. Clinical, electromyogram, genetic, and functional data were explored. The proband was an 11-year-old girl presented with progressive distal limb muscle weakness and atrophy due to peripheral motor neuropathy for 1 year. Another five members from three successive generations of the family showed similar symptoms during their first to second decades and demonstrated an autosomal dominant inheritance. The results of genetic testing revealed a novel c.383T>G mutation in the *GARS* gene in the affected individuals, showing apparent genetic cosegregation. Further bioinformatic analyses showed that the c.383T > G mutation resulted in L128R alteration in the second functional protein domain, and the mutation site was well conserved among different species. *In silico* analysis predicted that this mutation probably affected protein function. *In vitro*, this *GARS* mutation led to a different protein localization pattern than that of the wild-type enzyme. The study found a novel *GARS* mutation of c.383T > G causing dHMN-V with subcellular localization abnormity in a genetic cosegregation family. These findings broaden the mutational spectrum of *GARS*.

## Introduction

Distal hereditary motor neuropathy type V (dHMN-V), which is transmitted by autosomal dominant inheritance, can be caused by a heterozygous mutation in the glycyl-tRNA synthetase (*GARS*) gene (OMIM: 600287) on chromosome 7p14.3 or a heterozygous mutation in the *BSCL2* (OMIM: 606158) gene on chromosome 11q12.3. The clinical features of dHMN-V include muscular weakness and atrophy in the distal extremities, steppage gait, pes cavus, and absent or diminished deep-tendon reflexes. The upper limbs are predominantly affected. Because sensation is unaffected in dHMN-V, dHMN-V is distinct from Charcot-Marie-Tooth disease type 2D (CMT2D), which can also be caused by a *GARS* mutation (1). To date, 18 mutations in the *GARS* gene have been reported to be the underlying causes of dHMN-V or CMT2D. In this study, we report a novel mutation in the *GARS* gene that causes the autosomal dominant inheritance of dHMN-V in a Chinese family. Clinical, electromyogram, genetic, and functional data were explored.

## Methods

### Standard protocol approvals, registrations, and patient consents

Beijing Tiantan Hospital Ethics committee gave permission for our study, and written informed consent was obtained for all subjects.

### Subjects

The authors cared for the family for over 1 year; all medical care was within Tiantan Hospital. Results and notes were examined and recorded.

### Mutation analysis

**Figure 1:**
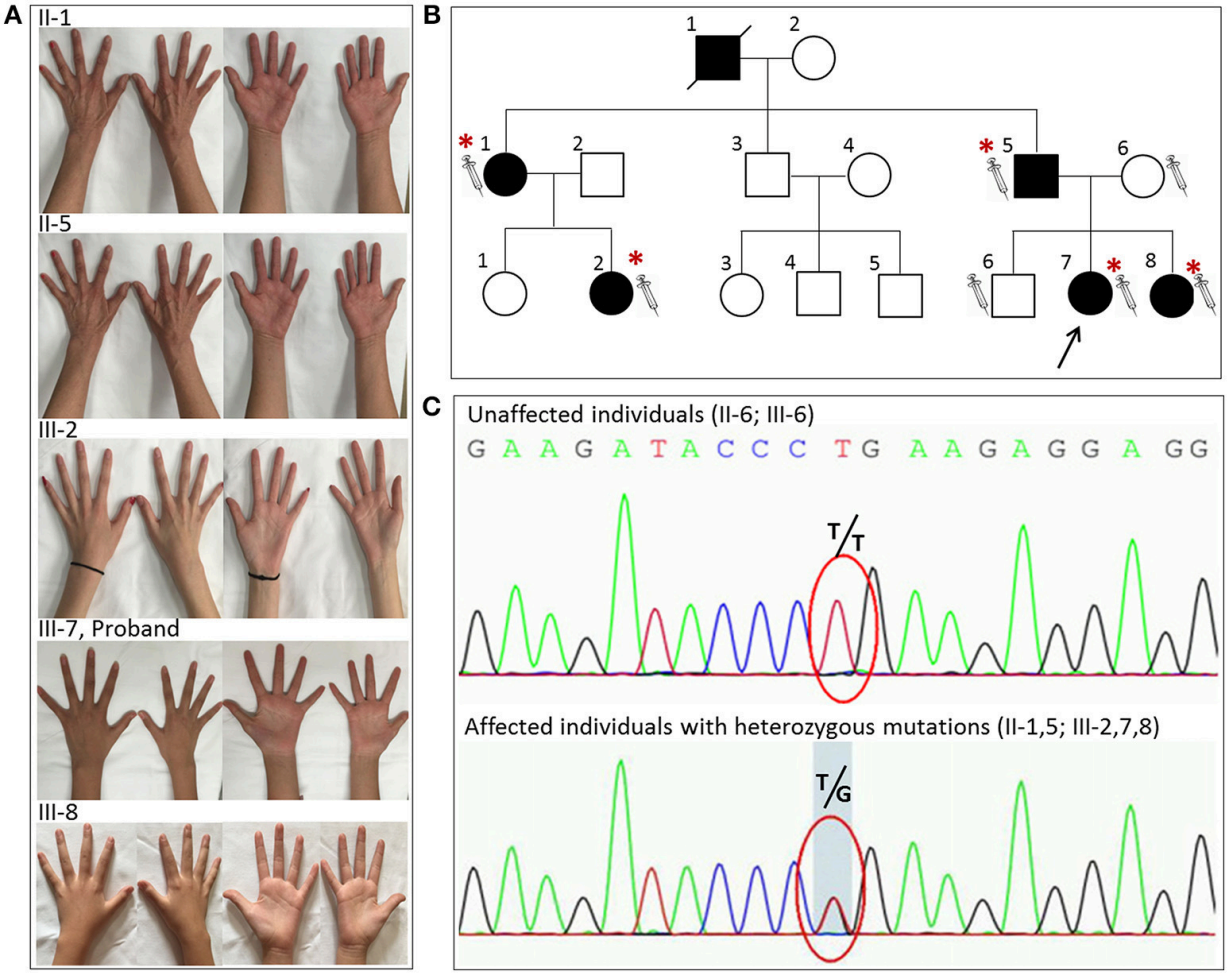
The novel glycyl-tRNA synthetase (*GARS*) mutation was detected in five individuals with distal hereditary motor neuropathy type V (dHMN-V). **(A)** Images of the hands of the patients with dHMN-V. II-1: The proband's aunt experienced weakness and muscle atrophy in the upper extremities bilaterally at 20 years of age. The lower extremities were less affected. II-5: The proband's father experienced weakness and muscle atrophy in the upper and lower extremities bilaterally at 16 years of age. III-2: The proband's cousin experienced muscle atrophy in the bilateral interosseous muscle at 6 years of age. III-7: The proband experienced weakness and muscle atrophy in the upper and lower extremities with pes cavus bilaterally at 10 years of age. III-8: The proband's young sister experienced weakness and muscle atrophy in the upper and lower extremities at 9 years of age. **(B)** The pedigree of the family with dHMN-V. Square = male; circle = female; diagonal black line = deceased individual; black filled symbol = clinically and electromyogram confirmed affected individual; empty symbol = clinically healthy relative; syringe symbol = blood sampled individual; asterisk: individual showing a *GARS* deleterious variant. **(C)** Chromatograms of the mutation sites confirmed by Sanger sequencing. Note that the five patients with dHMN-V had a novel homozygous mutation consisting of a guanine-to-thymine substitution at codon 383. Unaffected individuals had T/T, and the affected individuals with symptoms had the heterozygous mutation T/G at the corresponding codon. The red circle indicates codon 383.

The genomic DNA of the affected patient and the unaffected individuals (Figure 1) was extracted from peripheral EDTA-treated blood using the Blood Genomic Extraction Kit (Qiagen, Germany) and quantified using a NanoDrop 2000 unit (Thermo Fisher Scientific, Wilmington, DE). A gene capture panel analysis of the proband was conducted, including *SMN1, HSPB8, HSPB1, HSPB3, BSCL2, DNAJB2, GARS, REEP1, IGHMBP2, SLC5A7, DCTN1, MYH14, AARS, ASAH1, VRK1, EXOSC3, TRPV4, TFG, MAPT, DYNC1H1, BICD2, UBA1, APT7A, PLEKHG5, LAS1L, FBXO38, FBLN5, KLHL9*, and *MYH14*. These mutations were identified using next-generation sequencing. First, the variants were not selected if they appeared in the 1000 Genomes Project database with an MAF of >0.05. Then, the remaining variants were processed according to the dbSNP database. The single nucleotide polymorphisms (SNPs) and insertions/deletions (indels) were identified using the SOAPsnp and GATK programs. Subsequently, the reads were realigned to the reference genome (NCBI37/hg19) using BWA software.

### Expression analysis

*GARS*-EGFP fused genes cloned into the N3 vector. The c.383T>G mutation was introduced into the wild-type (WT) *GARS* sequence by site-directed mutagenesis using the QuikChange II Site-Directed Mutagenesis kit (Agilent), and a hemagglutinin tag added to the N-terminus. We transiently transfected 293T cells with the purified plasmid DNA 2 weeks later, and the stable transfected 293T cells were sorted. Expressions of WT and mutant proteins were analyzed by fluorescence microscopy.

## Results

### Clinical features of the family

**Figure 2:**
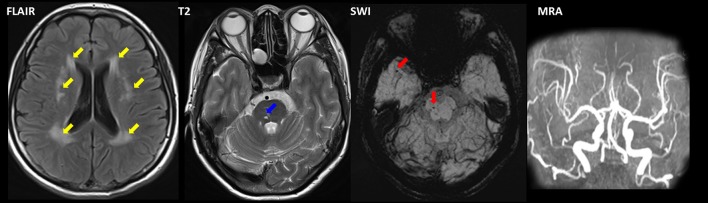
Magnetic resonance imaging of II-1 with combined dHMN-V and cerebral small vessel disease (CSVD). Fluid-attenuated inversion recovery (FLAIR) imaging revealed a hyperintensity (yellow arrow) surrounding the lateral ventricle and the bilateral centrum semiovale. Microbleeds (red arrow) and lacunar infarction (blue arrow) were present in the brainstem as observed using susceptibility weighted imaging (SWI) and T2. Magnetic resonance angiography (MRA) revealed normal intracranial arteries.

An 11-year-old girl presented with weakness and amyotrophy in the bilateral distal muscles for 1 year. Weakness was prominent in the upper extremities with writing difficulty. The patient had difficulty walking on her heels; however, she was still able to ambulate. No sensory, tremor, or bulbar symptoms were present. The physical examination revealed pes cavus in the bilateral feet and atrophy in the interosseous muscle and thenar eminence (Figure 1, III-7). The Medical Research Council scale values were grade 4 distally at the upper and lower limbs. The deep-tendon reflexes were hypoactive, and the Babinski signs were negative. The sensory examination was normal. The electrophysiological studies revealed a motor neuropathy in this individual with giant motor unit action potentials. The motor conduction showed a uniformly reduced velocity and CMAP amplitude in both the upper and lower extremities, with a more prominent reduction in amplitude. The sensory conduction studies were normal (Table 1, III-7). The family history showed similar symptoms in five other family members (Figure 1). Her grandparent, father, aunt, elder female cousin, and younger sister had similar symptoms at 19, 16, 20, 6, and 9 years of age, respectively. Her mother and brother did not display similar symptoms. This inheritance indicated automatic dominant inheritance, and this individual was clinically diagnosed with dHMN-V. Notably, her aunt (II-1) was also diagnosed with cerebral small vessel disease (CSVD) accompanied by dHMN-V. The brain magnetic resonance imaging of this patient showed key markers of CSVD as follows: white matter hyperintensities around the lateral ventricle and bilateral centrum semiovale; multiple lacunar infarctions and microbleeds in the brainstem; and relatively normal major intracranial arteries (Figure 2).

**Table 1:** Electromyogram studies in patients with GARS mutation.

		**III-7 (female)**		**II-1 (female)**	
Onset age (years)		10		20	
Examined age (years)		11		52	
**Side**		**Right**	**Left**	**Right**	**Left**
**MUP ANALYSIS**					
Tibial anterior muscle	Amplitude (mV)	2047 (522%↑)	2965 (802%↑)	O^*^	1117 (157%↑)
	Duration (s)	18.5 (56%↑)	19.5 (65%↑)	O^*^	18.4 (34%↑)
	PPP	90%	100%	O^*^	90%
Extensor digitorum	Amplitude	1170 (121%↑)	NA	2219 (289%↑)	NA
	Duration	15.8 (32%↑)	NA	17.5 (44%↑)	NA
	PPP	100%	NA	90%	NA
**MOTOR NCS**					
Median motor nerve	CMAP (mV)	4.2 (76%↓)	5.0 (72%↓)	NA	11.3
	MNCV (m/s)	34.0 (34%↓)	33.8 (48%↓)	NA	61
Ulnar motor nerve	CMAP (mV)	7.2 (57%↓)	8.9 (47%↓)	NA	11.9
	MNCV (m/s)	41.5 (38%↓)	37 (44%↓)	NA	52.1
Tibial nerve	CMAP (mV)	0.1 (99%↓)	NA	3.5 (73%↓)	3.4 (73%↓)
	MNCV (m/s)	O^*^	NA	O^*^	O^*^
Common peroneal	CMAP (mV)	O^*^	NA	NA	0.4 (92%↓)
	MNCV (m/s)	O^*^	NA	NA	26.5 (57%↓)
**SENSORY NCS**					
Median motor nerve	SNAP (μV)	46.3	NA	NA	27.9
	SNCV (m/s)	51.2	NA	NA	55.7
Ulnar motor nerve	SNAP (μV)	20.7	NA	NA	16.9
	SNCV (m/s)	54.9	NA	NA	53.3
Sural	SNAP (μV)	5.9	NA	NA	3.5
	SNCV (m/s)	51.8	NA	NA	56.7
Sup peroneal	SNAP (μV)	2.9	NA	NA	1.8
	SNCV (m/s)	57	NA	NA	57
Tibial	SNAP (μV)	1.6	NA	NA	0.6^&^
	SNCV (m/s)	38^$^	NA	NA	51

*MUP, motor unit potential; NCS, nerve conduction study; CMAP, compound muscle action potential; MNCV, motor nerve conduction velocity; SNAP, sensory nerve action potential; SNCV, sensory nerve conduction velocity; PPP, percentage of polyphasicity; NA, not available. O*

* *Unable to be detected*.

& *The low limit of normal range for amplitude of tibial SNAP is 0.5 μV*.

$ *The low limit of normal range for velocity of tibia SNCV is 35.1 m/s in the electrophysiological laboratory*.

### Genetic identification of *GARS* mutations

One variant in the *GARS* gene, i.e., c.383T>G, was identified in the targeted region capture sequencing data. Then, this mutation site SNP was validated by Sanger sequencing in the five affected patients and the two unaffected individuals from three successive generations of the family. The analysis of the available individuals in the family revealed that heterozygous mutations of c.383T>G in *GARS* consistently segregated with the disease, which followed an autosomal dominant mode of transmission. This mutation was present in all affected subjects (II-1, II-5, III-2, III-7, and III-8) and absent from the unaffected family members (II-6, III-6). This mutation has not been previously reported to worldwide databases for human genome polymorphisms (i.e., dbSNP135 and 1000 genome DB) or the Inherited Peripheral Neuropathies Mutation database (http://www.molgen.ua.ac.be/CMTMutations/mutations). Furthermore, this mutation was not detected in 200 normal controls or the ExAC database and could be classified as a “pathogenic variant” according to the American College of Medical Genetics and Genomics guidelines.

**Figure 3:**
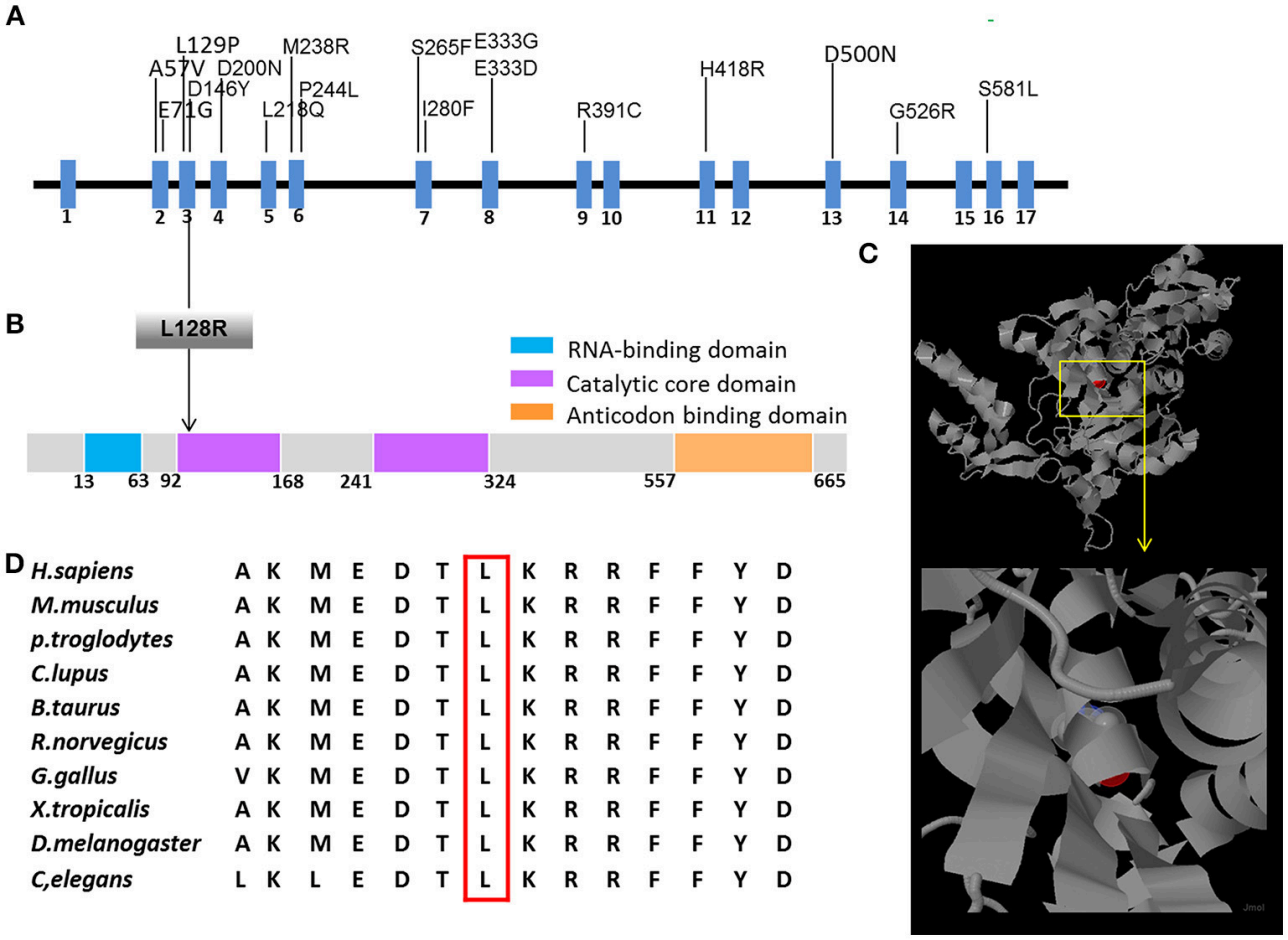
Schematic representation of *GARS* gene/protein and location of mutations. **(A)** cDNA structure of human *GARS* transcript and domain organization of human GARS protein. Above: mutations that have been previously reported in patients with CMT2D or dHMN. Below: positions of GARS protein changes (L128R) identified in patients with dHMN-V in this study. **(B)** The human GARS protein contains four functional domains, and the L128R mutation is located in the second catalytic domain. **(C)** The GARS monomer is displayed as a ribbon diagram. The positions of the amino acids that were substituted in the mutations found in the patients are highlighted in red. **(D)** Conservation analysis confirmed that the L128R mutation and surrounding amino acid sequences are well conserved among species.

This mutation resulted in the replacement of leucine with arginine at the Number 128 site of the amino acid sequence. We also obtained a 3D model of *GARS* from the RCSB protein data bank and found that this mutation in the GASR protein was located in the well-conserved catalytic domain. There have been no previous reports of dHMN-V or CMT2D caused by c.383T>G of the *GARS* gene worldwide. This mutation site and surrounding amino acid sequences were well conserved among different species (Figure 3). The *in silico* analysis using SIFT, MutationTaster, and Polyphen-2 predicted that this mutation likely affected protein function.

### Function identification of *GARS* mutations

**Figure 4:**
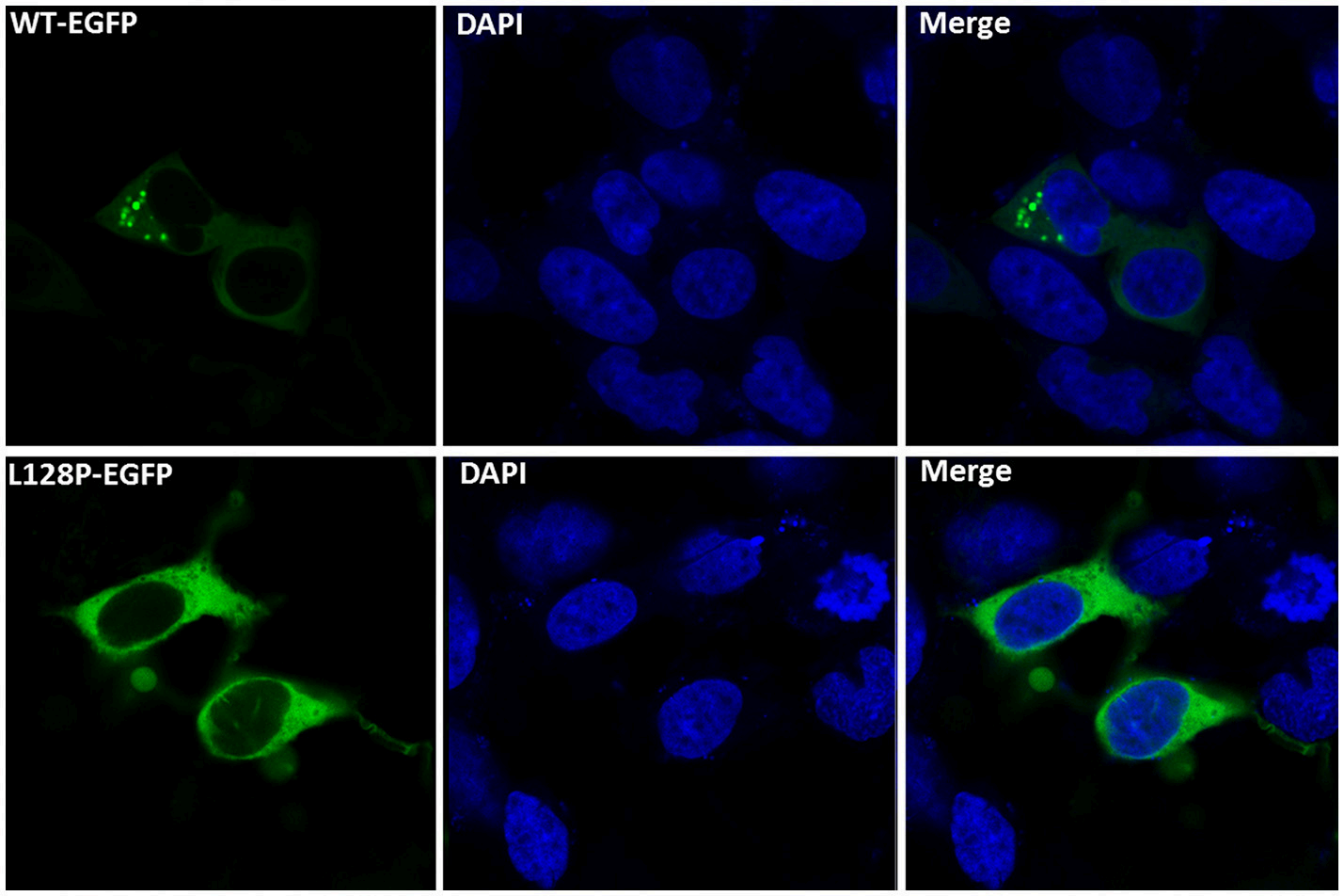
Functional analyses of disease-associated *GARS* mutations in an *in vitro* study. Wild-type (WT) and mutant *GARS–EGFP* were expressed in HEK-293T cells and examined by fluorescence microscopy. WT *GARS*–*EGFP*-associated granules within the cell body are indicated by white arrows.

To determine the function of the novel *GARS* mutations, we transfected HEK-293T cells with expression vectors containing WT or mutant *GARS* tagged with EGFP. The WT or mutant *GARS* genes were fused to the N-terminal of the *EGFP* gene and linked with the flexible sequence “GGGGS” to ensure that *GARS* and *EGFP* worked independently without affecting the protein topology structures and functions. WT *GARS-EGFP* associated with granules in the 40% cell of EGFP-positive 293T cells. In contrast, L128P *GARS-EGFP* did not associate with these granules (Figure 4). Thus, this *GARS* mutation resulted in a markedly different protein localization pattern than that observed with the WT enzyme.

## Discussion

In this study, we report the novel mutation c.383T>G in the *GARS* gene (p.L128R) in a Chinese family with dHMN-V. The family history confirmed autosomal dominant inheritance. The clinical features of the affected family members coincide with dHMN-V without sensation impairment. Genetic cosegregation was present in this family. Therefore, mutations of c.383T>G were first reported as pathogenic mutations with dHMN-V, because these mutations (i) were absent from all interrogated databases; (ii) showed apparent genetic cosegregation; (iii) were predicted to be pathogenic by at least three *in silico* prediction tools; (iv) affected highly conserved amino acids; and (v) caused a different protein localization pattern *in vitro* study.

The clinical features of dHMN-V mainly include distal limb muscle weakness and atrophy due to peripheral motor neuropathy. No sensory abnormality was detected in this family, which is inconsistent with CMT neuropathy. However, although dHMN-V has been previously characterized mainly by the distal degeneration of axons (NCV > 38 m/s), our case indicates that dHMN-V likely results in demyelination as well, but axonal degeneration is more profound (Table 1). Previous reports have identified a demyelination process in patients with dHMN-V, which is consistent with our findings (2–5). The disease onset in our cases ranged from 6 to 20 years of age, which is consistent with previous reports in which patients typically developed symptoms during adolescence or young adulthood.

Three genes have been reported to cause dHMN-V (i.e., *GARS, REEP-1*, and *BSCL-2*) (6). *GARS* contains 739 amino acids and functions in charging tRNAs with their cognate amino acids (7). This protein contains four functional domains; the L128R mutation resides in the second catalytic core domain, which is responsible for the ATP-dependent formation of the enzyme-bound aminoacyl adenylate. *GARS* mutations rarely cause hereditary neuropathy. No *GARS* mutations were found in a genetic screening of 109 Japanese patients with axonal CMT (8). In another study, mutations in 14 allelic genes associated with CMT disease were tested in more than 17,000 individuals, and genetic abnormalities were observed in 18.5% of the patients, whereas *GARS* mutations were observed in only 0.4% of the patients (9). To date, only18 mutations have been identified to be associated with CMT2D or dHMN (Figure 3) (2–5, 7–17). All reported *GARS* mutations were point mutations and occurred mostly in exons, with two exceptions that occurred in introns (9).

*GARS* mutations have also been found in other diseases, such as autism spectrum disorder, mitochondrial disease, and motor neuron disease (17–19). Interestingly, we found that CSVD was present in the patients with dHMN-V (II-1). Although no other authors have reported CSVD in this disease, a bilateral positive Babinski sign and hyperactive tendon reflexes were observed in some patients with dHMN-V, which could be explained by brain lesions (20–22).

*GARS* mutations are an uncommon cause of dHMN-V in China. This is the first study to report an association between the mutation of c.383T>G (p.L128R) and dHMN-V. These findings broaden the mutational spectrum of *GARS* and highlight the importance of considering *GARS* mutations to be causes of disease in patients with pure motor neuropathy.

## Author contributions

YY and ZZ conceptualized the study and acquired funding for this study. YF designed the study. YY, XY, BC, and HT collected the data. WL and XY analyzed the data. YF wrote the manuscript.

### Conflict of interest statement

The authors declare that the research was conducted in the absence of any commercial or financial relationships that could be construed as a potential conflict of interest.
